# Organization and Formation of the Crossed-Foliated Biomineral Microstructure of Limpet Shells

**DOI:** 10.1021/acsbiomaterials.3c00928

**Published:** 2023-11-22

**Authors:** Katarzyna Berent, Marta Gajewska, Antonio G. Checa

**Affiliations:** † Academic Centre for Materials and Nanotechnology, AGH University of Krakow, Krakow 30-059, Poland; ‡ Departamento de Estratigrafía y Paleontología, Universidad de Granada, Granada 18071, Spain; § Instituto Andaluz de Ciencias de la Tierra, CSIC−Universidad de Granada, Granada, Armilla 18100, Spain

**Keywords:** biomineralization, molluscs, calcite, material organization, crystallography, plywood structure

## Abstract

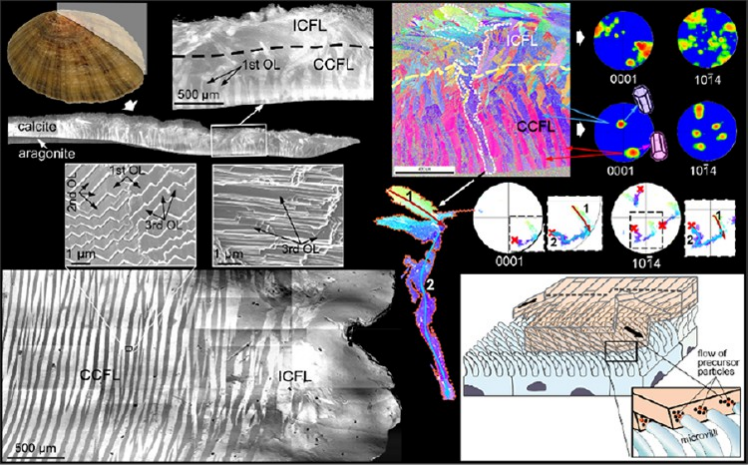

To construct their shells, molluscs are able to produce a large array of calcified materials including granular, prismatic, lamellar, fibrous, foliated, and plywood-like microstructures. The latter includes an aragonitic (the crossed-lamellar) and a calcitic (the crossed-foliated) variety, whose modes of formation are particularly enigmatic. We studied the crossed-foliated calcitic layers secreted solely by members of the limpet family Patellidae using scanning and transmission electron microscopy and electron backscatter diffraction. From the exterior to the interior, the material becomes progressively organized into commarginal first-order lamellae, with second and third order lamellae dipping in opposite directions in alternating lamellae. At the same time, the crystallographic texture becomes stronger because each set of the first order lamellae develops a particular orientation for the *c*-axis, while both sets maintain common orientations for one {104} face (parallel to the growth surface) and one *a*-axis (perpendicular to the planes of the first order lamellae). Each first order lamella shows a progressive migration of its crystallographic axes with growth in order to adapt to the orientation of the set of first order lamellae to which it belongs. To explain the progressive organization of the material, we hypothesize that a secretional zebra pattern, mirrored by the first order lamellae on the shell growth surface, is developed on the shell-secreting mantle surface. Cells belonging to alternating stripes behave differently to determine the growth orientation of the laths composing the first order lamellae. In this way, we provide an explanation as to how plywood-like materials can be fabricated, which is based mainly on the activity of mantle cells.

## 1 Introduction

The shells fabricated by invertebrates are composites of calcium carbonate crystals (predominantly calcite or aragonite) with a percentage of organic matter (1–12%),^1,2^ either occluded within the crystals or in the form of intra- or, more often, intercrystalline membranes.^3^ Shells are made of one or several layers, each characterized by three-dimensional arrangements of crystals with defined morphologies, termed microstructures. Within invertebrates, molluscs produce by far the largest repertoire of microstructures, followed at a considerable distance by brachiopods, polychaetes, and bryozoans. On the other end of the spectrum, groups, such as cirripeds, echinoderms, and scleractinian corals, produce only one or two basic microstructures. Molluscs can produce granular, prismatic, fibrous, laminar (foliated, nacre), plywood-like (crossed-lamellar, crossed-foliated), and helical microstructures with either calcite or aragonite. They form well over a dozen basic microstructural types with several subvarieties. The organomineral composite nature of microstructures provides them with exceptional biomechanical properties. Some of them, such as nacre and crossed-lamellar,^4−6^ are particularly strong and tough, whereas others are flexible in the native hydrated state, e.g., the columnar calcitic layers of pterioid bivalves.^7−9^ Accordingly, these biomaterials are a source of inspiration in biomimetics for the production of advanced functional materials. In this regard, it is necessary (1) to fully characterize the structure at different length scales and (2) to unravel the strategies used by the organisms to produce their microstructures. Molluscs make use of processes of crystal growth, and the self-organization of organic matrices, and their mantle cells are capable of recognizing the already laid-down structure (e.g., distribution of mineral versus organic substrates) and continue to secrete accordingly.^10^ These processes, combined or in isolation, produce strongly textured materials, however, with very different morphologies and arrangements from those of inorganic calcium carbonate aggregates. While there have been significant advances regarding the knowledge of microstructures such as nacre, columnar prismatic, and foliated microstructures, there is a very important microstructure that has remained particularly obscure, the crossed lamellar. This type constitutes one of the most hierarchically organized microstructures, consisting of first-, second-, and third-order lamellae (OLe; OL for singular, lamella). It is found mainly in aragonite, although a few gastropods, the patellogastropods, also produce a calcitic variety, the so-called crossed foliated. First OLe are wide lamellae (tens of micrometers) with irregular, jagged margins, which grow perpendicular to the growth surface. They can extend perpendicularly (radial) or parallelly (commarginal) to the shell margin. The aragonitic variety consists of fibers (third OLe), which arrange in sheets (second OLe), while the calcitic type is made of long laths with arrowhead endings (third OLe) that organize into folia (second OLe). In both cases, the second and third OLe dip in opposite directions in alternating first OLe, such that orientations are identical every two first OLe. Since the aragonitic crossed-lamellar microstructure was defined by Bøggild,^11^ many authors have dealt with its structure,^12−16^ and crystallography.^15,17−20^

The calcitic crossed-lamellar microstructure remained relatively neglected until the extensive study of MacClintock^21^ on patellogastropods, who renamed it as crossed-foliated, according to the foliated aspect of the second and third OLe. The term crossed-lamellar remained for the more fibrous aragonitic variety. The aragonitic crossed-lamellar microstructure is by far the most successful microstructure in molluscs. It is prevalent in gastropods, bivalves, and polyplacoporans and the only microstructure produced by scaphopods. It was also present in the fossil molluscan class Rostroconchia,^22^ and in the phylum Hyolitha.^23^ The crossed-lamellar microstructure arose independently in different classes. It is absent in cephalopods, monoplacophorans, and the spicule-producing aplacophorans. Due to this and to its excellent biomechanical properties, particularly toughness,^6,24^ the crossed-lamellar microstructure has been the focus of a myriad of studies, and presently there is a great deal of information on its morphology and crystallography.

The crossed-foliated microstructure, on the contrary, is restricted to the gastropod family Patellidae (subclass Patellogastropoda), where it is usually the second layer from the exterior.^21^ A seemingly crossed-foliated material was described in some lower Jurassic Gryphaeidae^25^ and in upper Jurassic Oxytomidae,^26^ although these assignments are difficult to verify in the absence of 3D views of the material. With regard to the Patellidae, very few studies provide scanning electron microscopy (SEM) evidence on the organization of the material.^27−30^ In addition, nothing is known about its crystallography. Given its interest as a hierarchically organized material, this study focuses on the organization and crystallographic texture of the crossed-foliated microstructure. We develop a crystallographic model for this microstructure and show that, contrary to its aragonitic counterpart, the crossed-lamellar microstructure organizes progressively from a poorly organized crossed-foliated external layer. We hypothesize that such a progressive organization is obtained through a combination of crystallographic processes and mantle cell activity. Our study provides insight as to how crossed-foliated or plywood-like biomineral structures can be organized.

## 2 Experimental Section

Seven living specimens of the limpet *Patella caerulea* were sampled in the locality of Almuñécar (Mediterranean, SE Spain), whereas four empty shells of *P. rustica* and four of *P. depressa* came from Avilés (Atlantic, NW Spain). Shells were washed with distilled water and air-dried at room temperature. Five shells of *P. caerulea* were cleaned with commercial bleach (approximately 5% active chlorine) for 4–5 min. Three to four bits of the internal surfaces of two shells of *P. caerulea* and one shell of each of the two other species were mounted on SEM stubs, carbon-coated (Emitech K975X carbon evaporator), and observed in field-emission SEM (FESEM) equipment Zeiss Auriga and FEI QemScan 650 F at the Center for Scientific Instrumentation (CIC) of the University of Granada (UGR), Spain, and in the FESEM FEI Versa 3D of the Academic Centre of Materials and Nanotechnology (ACMiN), Krakow, Poland.

Atomic force microscopy (AFM) observations were done on inner surfaces of shell fragments of *P. caerulea*, previously ultrasonicated. We used an AFM Park Systems NX20 (CIC, UGR) equipped with a cantilever ACTA (K = 40 N/m, F = 280 kHz) (CIC, UGR). We recorded height, amplitude, and phase signals in tapping mode. Images were obtained with Smart Scan v12 and analyzed with XEI 4.3 software (Park Systems).

The mantle of a living specimen of *P. caerulea* was excised and fixed in a mixture of 2.5% glutaraldehyde buffered with sodium cacodylate (0.1 M, pH 7.4) for 48 h at 4 °C and postfixed in OsO_4_ (2%) for 2 h at 4 °C. The tissue was later embedded in epoxy resin, Aname Epon 812 (EMS). Ultrathin sections (0.1 μm) were stained with uranyl acetate (1%) and lead citrate. They were later carbon-coated and observed with transmission electron microscopy (TEM) Zeiss Libra 120 Plus instrument of the CIC (UGR).

Electron backscatter diffraction (EBSD) analysis was performed to identify the crystal structure. Shell fragments of the three species were mounted in epoxy, sectioned perpendicular to the shell surface, ground, and polished following a standard metallographic preparation route: mechanical polishing by silicon carbide papers with grit sizes of 320, 500, 800, 1200, 2000, and 4000, followed by 3, 1, and 0.25 μm diamond suspension. Final polishing with colloidal silica for 1 min using a Struers Tegramin-25 automatic polisher ensued. In this way, two sections of each species were mapped. Other maps were performed directly on the internal shell surfaces of *P. depressa* and *P. caerulea* without further polishing, despite which they rendered useful information and allowed us to relate the shapes of calcite laths to crystallographic orientations. EBSD analysis was carried out with an FEI Versa 3D FESEM, equipped with an Oxford Instruments Symmetry S2 camera. EBSD maps were collected by applying an acceleration voltage of 12 kV under low vacuum (40 Pa of H_2_O). Data were analyzed with the Data Collection v.7.3 and Aztec 6.0 software. To calculate the number of grains, the grain boundaries were defined where pixel-to-pixel misorientation was above a critical misorientation angle, set at ≥15°. Multiple of uniform density (MUD) provides an indication of the strength of the texture. A MUD of 1 indicates random orientation, while high MUD values (600–700) indicate a high, single-crystal-like co-orientation. The focused ion beam (FIB) lift-out method was used to prepare two ultrathin cross-sectional lamellae of the concentric crossed-foliated microstructure of*P. caerulea* using a FEI Quanta 3D 200i dual beam FIB/SEM. The TEM lamellae were milled down to ∼50 nm thickness using 2 kV. This removed the beam surface damage. Bright-field imaging and selected area diffraction (SAED) analysis of the two lamellae were performed using an FEI Tecnai TF 20 X-TWIN TEM operated at 200 kV. All the above equipment is housed in the ACMiN.

## 3 Results

### 3.1 Shell Microstructure

**Figure 1:**
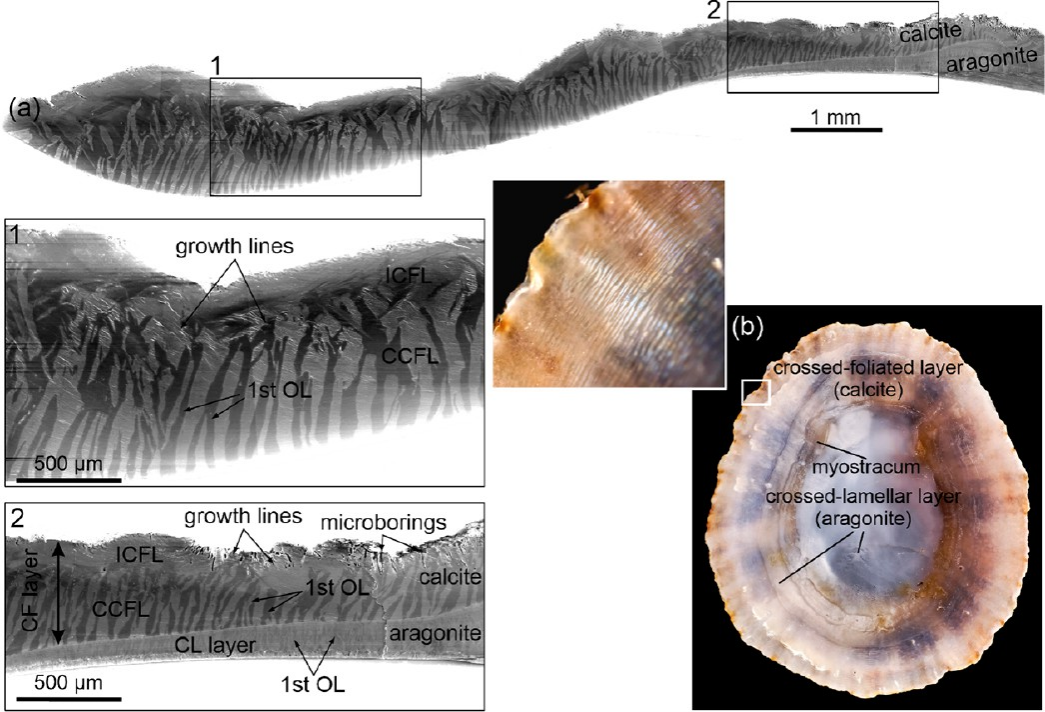
General shell structure of the studied species of *Patella*. (a) Radial section through the shell of *P. depressa*, indicating the calcitic and aragonitic shell layers. Details of both can be seen in insets 1 and 2. (b) View of the interior of the shell of *P. caerulea*, showing the distribution of shell layers. The inset shows the commarginal distribution of first OLe. CCFL, ICFL: concentric crossed-foliated layer, irregular crossed-foliated layer; CL: crossed-lamellar.

The general shell structures of the three species studied are similar. There is a thick outer calcitic shell portion, made of crossed-foliated material, internal to which there is an aragonitic crossed-lamellar shell portion (Figure 1a), crossed by the myostracum. The calcitic shell portion thickens in the direction toward the margin, and, contrarily, the crossed-lamellar layer wedges out in the same direction. The calcitic portion consists of first OLe with a commarginal distribution, which, in the radial section of Figure 1a, is cut transversely. Their outlines become better defined toward the shell interior and are oriented at a high angle to the growth lines (Figure 1a, insets 1 and 2). Toward the apex, the shell is usually bored progressively deeper interiorly by endolithic microorganisms. The first OL of the underlying crossed-lamellar (aragonitic) layer is also cut transversely, and similar to the crossed-foliated layer, they are at a high angle to the growth lines and to the internal shell surface (Figure 1a, inset 2). In optical views of the internal shell surface, the outer calcitic portion appears slightly translucent, and at a certain distance from the margin, the distribution of commarginal first OL is clearly marked by differences in reflectance (Figure 1b, inset). The more internal crossed-lamellar material appears gray and dull. It occupies the rest of the shell toward the interior, except for the intercalation of the myostracum (Figure 1b).

**Figure 2:**
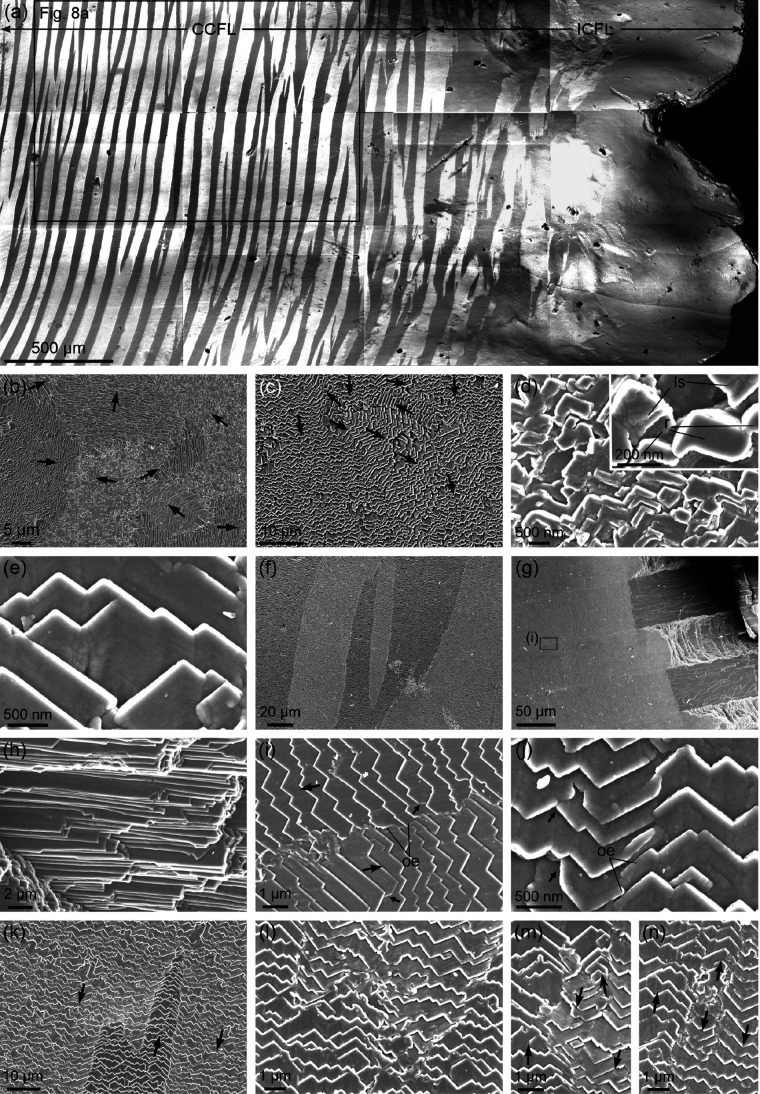
Details of the crossed-foliated microstructure. (a) View of the internal shell surface of *P. rustica*. The alternating first OLe can be differentiated due to orientation contrast. They are irregular toward the margin (irregular crossed-foliated [ICF] layer, right side) and become commarginal toward the interior (concentric crossed-foliated [CCF] layer). The framed area is reproduced in black and white in Figure 8a. (b, c) Irregular distribution of first OLe of the ICF layer close to the margin of *P. caerulea*. Also, note the differences in shape and size. (d) Details of the third OL at the margin of *P. caerulea*. They are particularly thick and have coarsened edges. The inset is a detail. The rhombohedral surfaces looking toward the observer (r) are particularly smooth, compared to the lumpy aspect of the rest of the growth surfaces (ls). (e) Details of the third OLe of the ICF layer of *P. caerulea* in an area similar to those in b and c. They are identical to those of the CCF layer (e.g., i, j). (f) Distribution of first OLe on the internal shell surface of *P. caerulea* at the transition between the ICF layer and the CCF layer. (g) View of the CCF layer of *P. rustica*. The fracture on the right allows us to observe the changing inclinations of the second and third OLe in alternating first OLe. (h) Fracture through a first OL of *P. rustica*, showing the extent of the third OLe. (i) Detail of g (framed), at the contact between adjacent first OLe in *P. rustica*. Note slightly overlapping edges (oe) at the contact between second OLe dipping in opposite directions. Small arrows indicate initiating second OLe. (j) Detail of a similar contact in *P. caerulea*. Some overlapping edges (oe) and initiating second OLe (arrows) are indicated. (k) Two-tailed end of a first OL of the CCF layer of *P. caerulea* (central part of the image). (l) Close-up of a similar case in *P. caerulea*. Note irregularities at the very tips. (m, n) Two cases of divergence of first OLe in *P. caerulea*, beginning with a single third OL. In n, the divergence happens from a single spot. Arrows in b, c, i (large arrows), k, m, and n indicate the growth directions of the third OLe.

The distribution pattern of the first OLe of the crossed-foliated layer becomes clearer in SEM views. In general, they tend to be irregular close to the edge; however, directly toward the interior, they become commarginal (Figure 2a). Furthermore, the marginal first OLe are irregular in shape and very uneven in size (Figure 2a–c). Their widths may reach >200 μm locally. Their constituent laths (third OLe) have even orientations within each lamella but can take any orientation with respect to neighboring lamellae (Figure 2b,c). At the very margin, the third OLe are sometimes particularly thick (up to 0.5 μm), and their terminal edges coarsen and develop rhombohedral facets (Figure 2d). These are much smoother than the rest of the surfaces, which have a typical lumpy aspect. Directly adjacent to the margin toward the interior, the third OLe become thinner (0.2–0.3 μm) and arrange into more continuous second OLe (Figure 2c,e). Given the unevenness in size and orientation of the first OLe, we will call this layer an irregular crossed-foliated (ICF) layer (Figure 2a).

After a brief transitional stage (Figure 2a,f), the first OLe become commarginal. They constitute the CCF layer. Their widths are not consistent laterally and frequently wedge out (Figure 2a), with the consequent fusion of alternating first OLe containing evenly oriented second and third OLe (Figure 2a), in a sort of zebra pattern. Their maximal widths ranged from 50 to 70 μm (Figure 2a,g). The third OLe have widths between <200 nm and ∼2 μm, and, in fracture, they extend toward the shell interior for unknown distances (at least tens of micrometers) (Figure 2g,h). They arrange into continuous second OLe, although these may not extend along the entire thickness of the first OLe (Figure 2i). Third and second OLe dip with similar angles but in opposite directions in alternating lamellae (Figure 2g,i–k). The junctures between the third OLe of adjacent first OLe are tight, and tend to interpenetrate slightly (Figure 2i,j). In the radial direction, the boundaries of the first OLe are not simple and may consist of several branches, giving it a jagged appearance (Figure 2k,l). The tips may consist of just one or a few laths (Figure 2l–n). AFM observations of the main surfaces of laths of the CCF layer of *P. caerulea* revealed a marked surface nanoroughness, consisting of lumps between 10 and <100 nm (Figure S1). Lumps superimpose and merge with each other, thus having undefined boundaries. Phase images reveal the presence of two phases, a predominant light phase and a darker phase distributed around the boundaries between the lumps (Figure S1, phase images). Only the terminal arrowheadlike faces and their margins appear smoother.

The distribution of the ICF and CCF layers is also evident in the longitudinal section of Figure 1a, insets 1 and 2. In the CCF layer, the transversely sectioned first OLe are approximately parallel but unevenly oriented in the ICF layer. The first OLe increase in thickness from the juvenile (Figure 1a, inset 2) to the subadult (Figure 1a, insert 1).

### 3.2 EBSD Analysis

**Figure 3:**
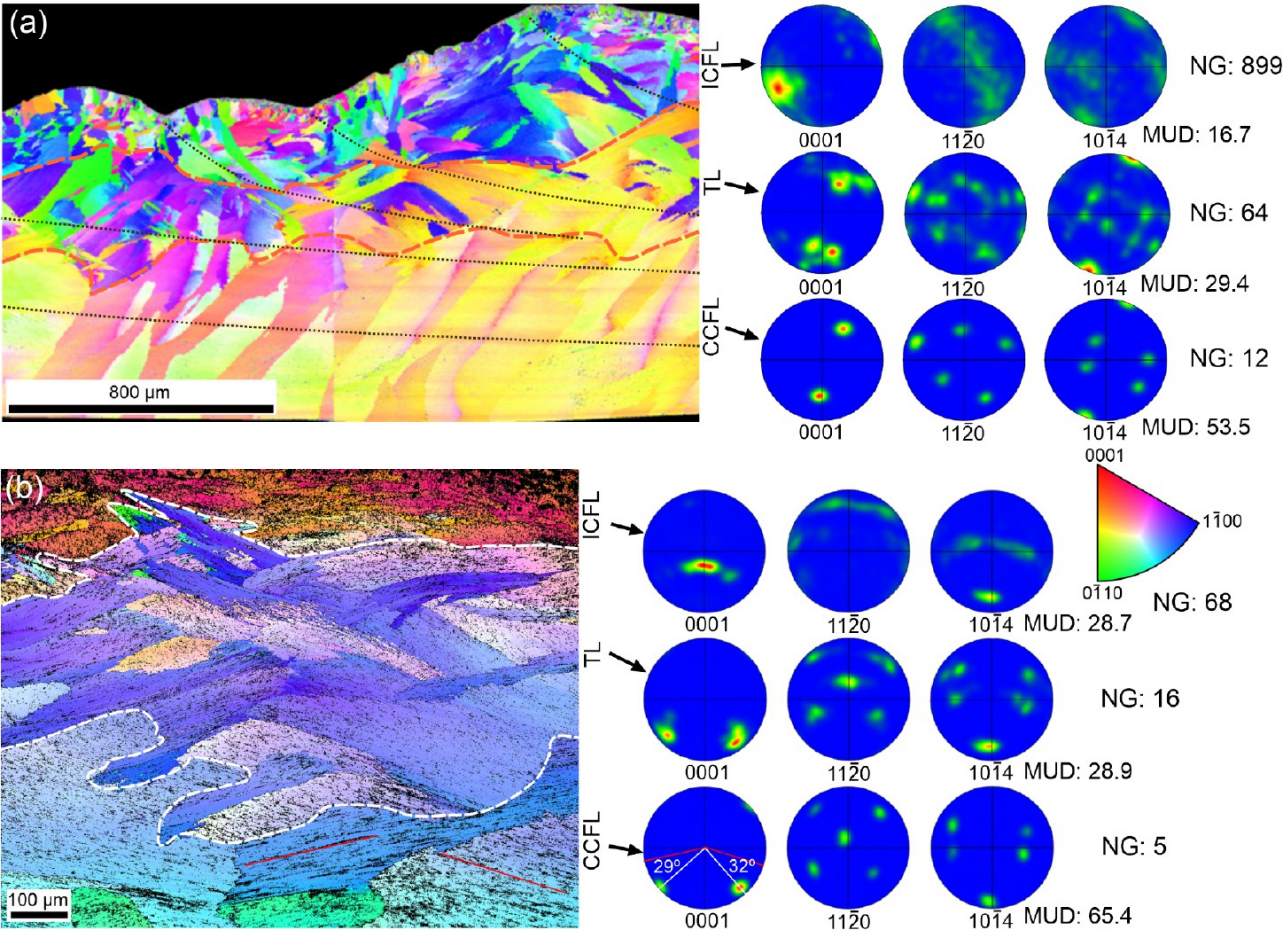
IPF maps and pole figures of the layers distinguished within the shells of Patellidae. (a) Radial section of *P. depressa*, with growth lines indicated (black dotted lines). (b) Commarginal section of *P. caerulea*. The red lines at the bottom of the image are the approximate orientations of the second OLe. CCFL, ICFL, TL: concentric cross-foliated, irregular cross-foliated, transitional layers. NG is the number of grains for each layer. The color triangle is the color key for orientations.

Inverse pole figure (IPF) orientation maps done on radial sections provide additional insight into the organization of the material. The outer shell layer is made of elongated lamellae with irregular sizes and shapes, which are mainly inclined in the growth direction and at a high angle with respect to the growth lines (Figure S2). In other cases, the lamellae do not display consistent orientations (Figure 3a). Orientation maps display a varied range of colors indicative of varied orientations (Figures 3 and S2). We can assimilate this layer to the ICF layer identified with SEM observations. Rarely, a thin (∼10–20 μm thick) outermost layer made of very small and short lamellae can be observed (Figure 3a).

Deeper within the shell, this ICF layer is transformed into the CCF layer. The change takes place through a transitional layer, which can have a more (Figures 1a and S2a) or less reduced thickness (Figure S2b). From the ICF layer to the CCF layer, the number of first OLe decreases while their sizes increase. Values for the number of grains across the different layers are listed in Figures 3 and S2. At the end of the transitional layer, the first OLe become neatly defined and acquires a homogeneous orientation at a high angle to the growth lines and to the internal shell surface. Across the CCF layer, the first OLe display irregularities in thickness similar to those observed in the plan view: branching, widening, thinning, and disappearance. Maximal thicknesses are on the order of 200 μm (e.g., Figure 3a). Alternating first OLe display alternating colors, which indicate the existence of two main orientations. Within each individual lamella, the gradual color changes indicate internal misorientations in both the longitudinal and transversal directions. Extreme values are in the order of 20° in the direction of the lamella thickness and 50° in the growth direction.

We tentatively estimated the boundaries between the ICF layer, the transitional layer, and the CCF layer and plotted their pole figures (Figures 3 and S2). In all cases, the material displays a certain degree of order. The ICF layer demonstrates an overall orientation, with the *c*-axis (the 001 maximum) pointing in the growth direction and at a high angle to the growth lines. There are ill-defined 104 and 110 maxima, such that the texture can be qualified as very weak (Figure 3a) to weak (Figures 3b and S2) sheet texture. The internal CCF layer, on the contrary, has well-defined maxima for all axes (strong sheet texture) and a complete change in the distribution of maxima. There are two maxima for the *c*-axis, which are aligned parallel to the elongation of the first OLe at an angular distance of ∼90°. Each maximum corresponds to one set of alternating first OLe. The 110-pole figure displays five maxima, with one of them being common to both sets of first OLe and oriented perpendicular to the elongation of the first OLe. Finally, out of the five 104 maxima, there is one that is common to both sets of first OLe. It is placed close to either the N or S pole of the pole figure and is aligned with the two 001 pole maxima. The position of the common 104 maximum can be traced from the ICF layer to the CCF layer. The transitional layer has a distribution of maxima closer to that of the CCF layer (Figures 3a and S2a), or intermediate between the ICF layer and the CCF layer (Figure S2b). MUD values indicate that the degree of ordering is the highest in the CCF layer in all cases. The transitional layer values can be higher (Figure 3a), similar (Figure S2a), or lower (Figure S2b) than those of the ICF layer, although this is possibly partly dependent on how the transitional layer is defined.

In a commarginal section (approximately parallel to the first OLe) (Figure 3b), the distribution of maxima is as described above, although rotated by 90° around the vertical axis of the pole figures (the line joining the N and S poles). In the example in Figure 3b, the second OLe are inclined by ∼14° in both directions; that is, their angle with the growth surface is ∼76°. Other measurements on the same species provide inclinations of 18° and 21°. Interestingly, the two 001 maxima are placed in the direction of inclination of the corresponding second OLe, although at a higher angle. The two 001 maxima and the common 104 maximum are located along the same meridian. As usual, the MUD values are similar for the ICF layer and transitional layer and are the highest for the CCF layer.

**Figure 4:**
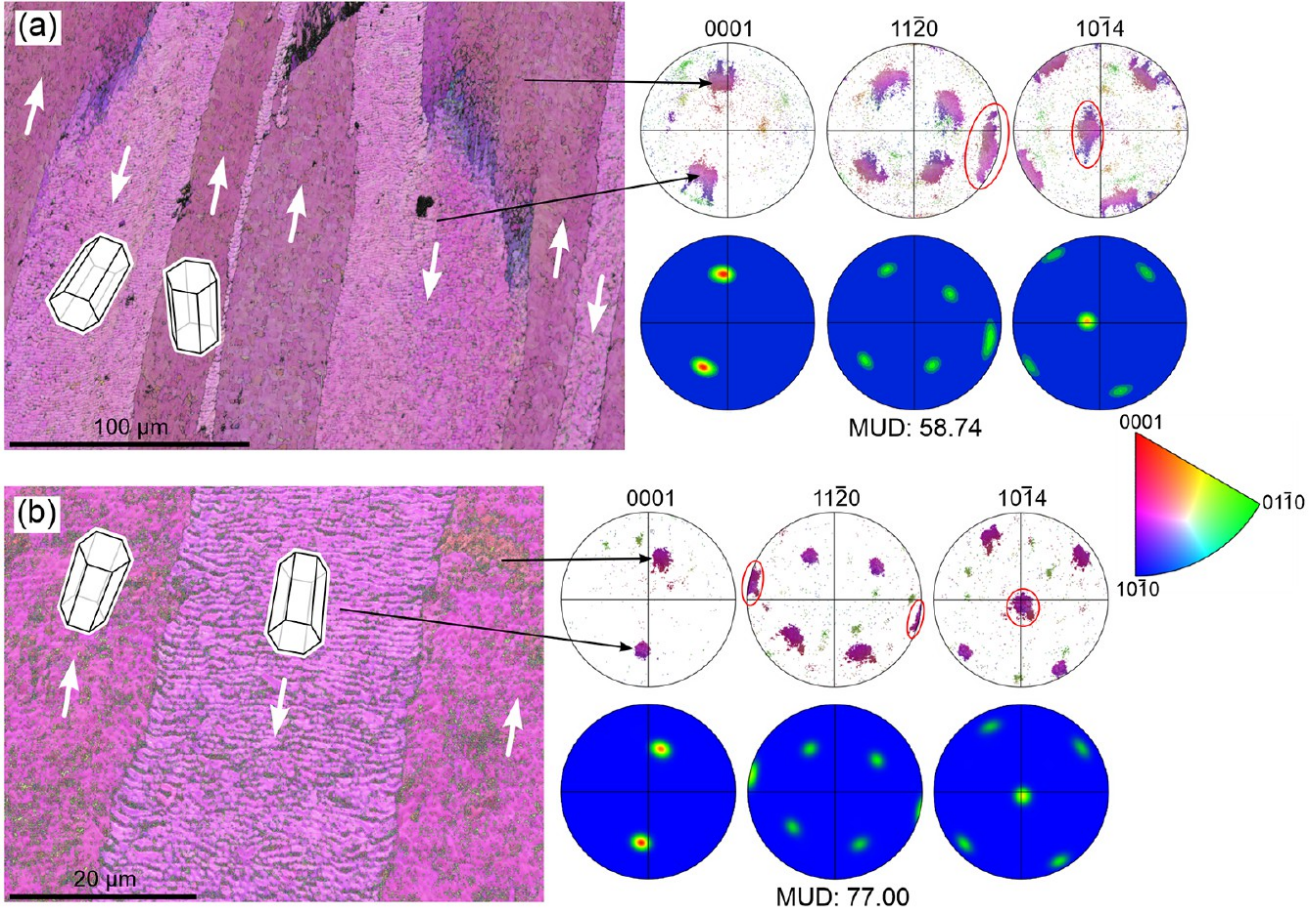
IPF maps, at different magnifications, done directly on the growth surface of *P. depressa*, together with the corresponding raw and contour pole figures (a, b). The 001 maxima are joined to their corresponding set of the first OLe with thin arrows. The overlapping 110 and 104 maxima are encircled with red ovals. Thick arrows indicate the growth directions of the third OLe. The color triangle is the color key for orientations.

In order to better define the crystallography of the CCF layer, we have mapped directly the internal unpolished surfaces of the shells, such that the orientation of the second and third OLe can also be appreciated (Figure 4). On the maps, alternating first OLe come in slightly different colors, and those of the same set (every two lamellae) come in similar colors, indicating different and similar orientations, respectively. There are two 001 maxima aligned with the axis of elongation of the first OLe, at angular distances of ∼45° from the center, each corresponding to one set of first OLe. Within each set, the c-axis is inclined in the same direction as the laths (i.e., opposite to the growth directions of laths; large arrows and unit cells in Figure 4). One of the 110 maxima is coincident or almost coincident for both sets of lamellae. It is placed perpendicularly to the elongation of the first OLe. There are two 104 maxima, one for each set of first OLe, placed centrally, which, similarly to the 110 maxima, overlap completely or to a high extent. The MUD value is lower in Figure 4a than that in Figure 4b, with the first covering a much greater area. This indicates that the spread of poles increases with the size of the mapped area.

**Figure 5:**
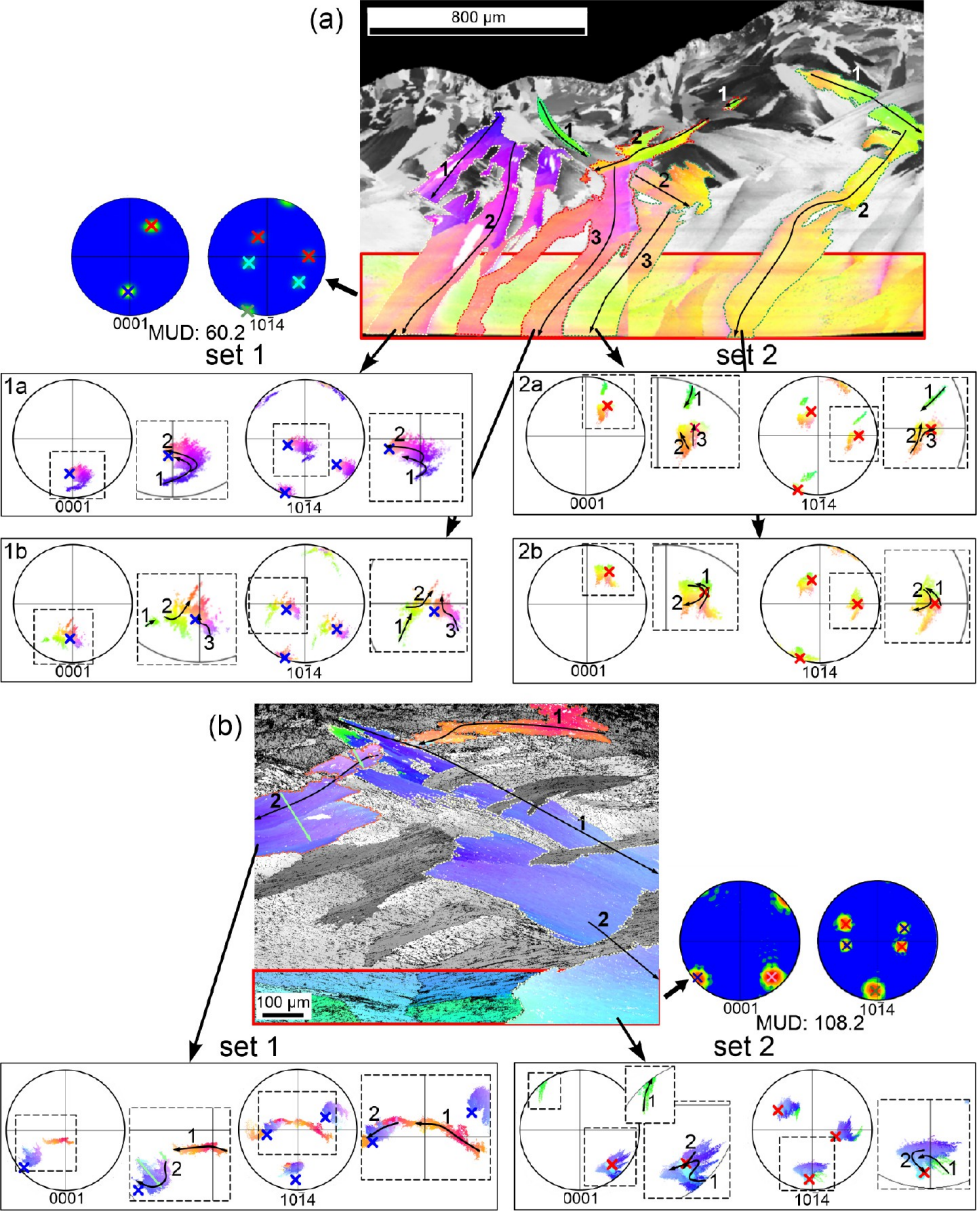
Growth trajectories of the first OLe selected on the orientation maps of Figure 3. (a) *P. depressa*. Two first OLe per set have been selected. (b) *P. caerulea*. One first OL per set has been selected. Pole figures next to the orientation maps correspond to the rectangular area close to the growth surface. Positions of maxima for the two sets of first OLe are indicated with red, blue, and green crosses. Individual scatter pole figures for the selected lamellae are provided (color crosses indicate the positions of the maxima close to the growth surface for the corresponding set of first OLe). The trajectories (black arrows) are indicated both on the first OLe and on the magnified areas of the pole figures. These consist of two to three segments. The light blue arrows in both the lower map and its 001 pole figure (magnified area) indicate the high misorientation of the *c*-axis across the thickness of the first OL (∼30°). The color key for orientations is provided in Figure 3.

An obvious feature in all color maps is that most of the individual first OLe of the CCF layer can easily be traced to the transitional layer and sometimes even to the outer ICF, also according to continuous color gradients in the EBSD color maps. When two supposedly continuous lamellae are not in contact due to the 2D sectioning or when the lamella is interrupted by another lamella, we have used the continuity of the trend of the misorientation plot as additional evidence of crystallographic continuity (Figure S3). A growth trajectory (usually consisting of several segments) is provided for each selected first OL on the orientation map. We also plotted the 001 and 104 raw pole figures for the individual first OLe, together with the pole movement along the growth trajectory traced on the IPF map. In this way, we can observe how the *c*- and *a*-axes of a particular first OLe migrate with growth. We have also determined the positions of the 001 and 104 poles on the area closest to the internal shell surface (Figures 5 and S4), where the coorientation (MUD values) is maximal. In the examples depicted in Figures 5 and S4, we can observe how the 001 and 104 poles migrate progressively, starting from very disparate positions of the pole figure and ending close or very close to the position of the maxima corresponding to their particular set of first OLe (crosses on pole figures in Figures 5 and S4). The total angle covered by the trajectories on the pole figures is high, sometimes above 90° (e.g., Figure 5b, set 1, both pole figures; Figure S4b, set 2, all pole figures).

### 3.3 TEM Measurements

**Figure 6:**
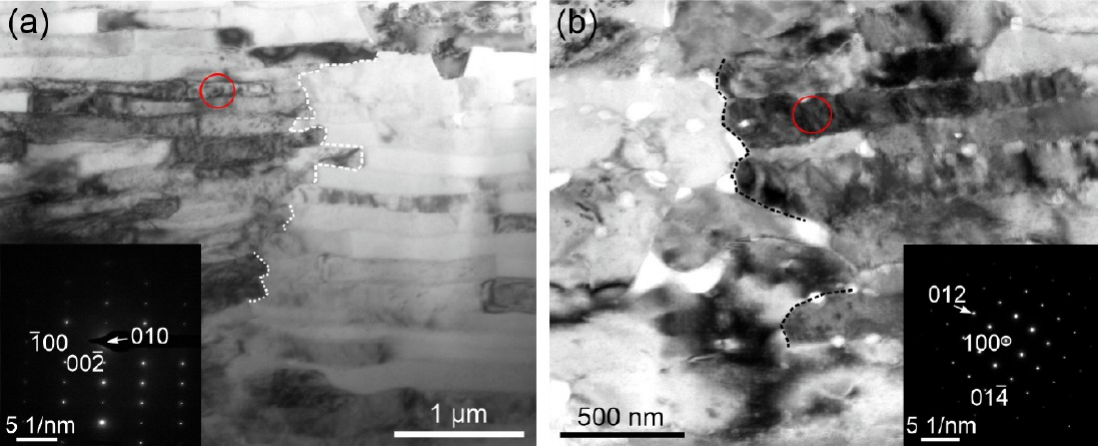
Views of the two lamellae prepared with FIB of the CCF layer of *P. caerulea* (a, b). The red ovals indicate the areas from which the SAED patterns (inserts) were taken. The boundaries between first OLe have been depicted with broken lines (based on dark field images, not shown). Due to the inclination of the laths (∼45°), the observed thickness is larger (by ∼1.4 times) than the actual thickness. The SAED patterns recorded from a and b indicate that the laths are seen along the [010] and [100] directions, respectively.

The two TEM lamellae of the CCF of *P. caerulea* examined were prepared by FIB-SEM perpendicular to the internal shell surface and toward the elongation of the first OLe. In this way, the laths were cut parallel to their width at an angle of ∼45° to their growth axis. TEM bright field images revealed that, in depth, the boundaries between juxtaposed laths belonging to adjacent first OLe could easily shift laterally, producing a jagged boundary (Figure 6, broken lines). The measured thicknesses of lamellae varied between ∼60 and ∼150 nm (average of ∼110 nm). For single lamellae, the thickness may also vary laterally. The lath orientations obtained from the SAED spot patterns (insets in Figure 6a,b) were along the [010] and [100] zone axes in Figure 6a,b, respectively, which is congruent with the EBSD data.

## 4 Discussion

### 4.1 Shell Microstructure

The crossed-foliated microstructure is a hierarchical material composed of calcite laths (third OLe) arranged in planes (second OLe), in turn packed into big first OLe perpendicular to the shell surfaces. The evenness in orientation and size of the latter sets the difference between the ICF and the CCF layers.

In his extensive review of shell microstructures, MacClintock^21^ established a nomenclature for different shell layers in patellogastropods (limpets). He named the layers from M-2 to M+4, with reference to the myostracum (layer M). He grouped the limpets according to the number of layers and their microstructures into 16 groups, and included two of the species we have studied (*P. caerulea* and *P. lusitanica*, presently a synonym to *P. rustica*(31)) within Group 8, characterized by M+3 = radial crossed-foliated, M+2 = concentric crossed-foliated, M+1 = concentric crossed-lamellar, M, myostracum, M-1 = radial crossed-lamellar and/or complex crossed-lamellar, and M-2 = irregularly foliated to radial crossed-foliated. Our third species, *P. depressa*, not examined by MacClintock, has exactly the same distribution of calcitic layers (Figures 1a, 3a, and S2a). Here, we have paid attention to MacClintock’s M+3 (our ICF) and M+2 (CCF) layers, although we call the former as irregular, instead of radial crossed-foliated layer, in view of the irregularity in size and orientation of the constituting lamellae (e.g., Figure 2).

Close inspection of the CCF microstructure with AFM revealed the surface nanoroughness of laths (third OL) (Figure S2), together with the presence of a majority light phase and a residual dark phase. This biphase nanoroughness is typical of most biocrystals,^32,33^ and the two phases are presently interpreted and crystalline (light phase), and amorphous (dark phase), the latter consisting of amorphous calcium carbonate together with biomolecules.^34,35^ Nanoroughness vanished close to and at the terminal arrowhead faces, for crystallographic reasons (see next subsection).

### 4.2 Crystallography of the CCF Microstructure

The IPF maps done on both radial and commarginal sections reveal that the ICF microstructure has a fiber texture, whereas the CCF microstructure has a sheet texture. The texture of the CCF layer is also stronger according to the MUD values (Figures 3 and S2). The change from one texture to the other takes place across a thick transitional layer.

**Figure 7:**
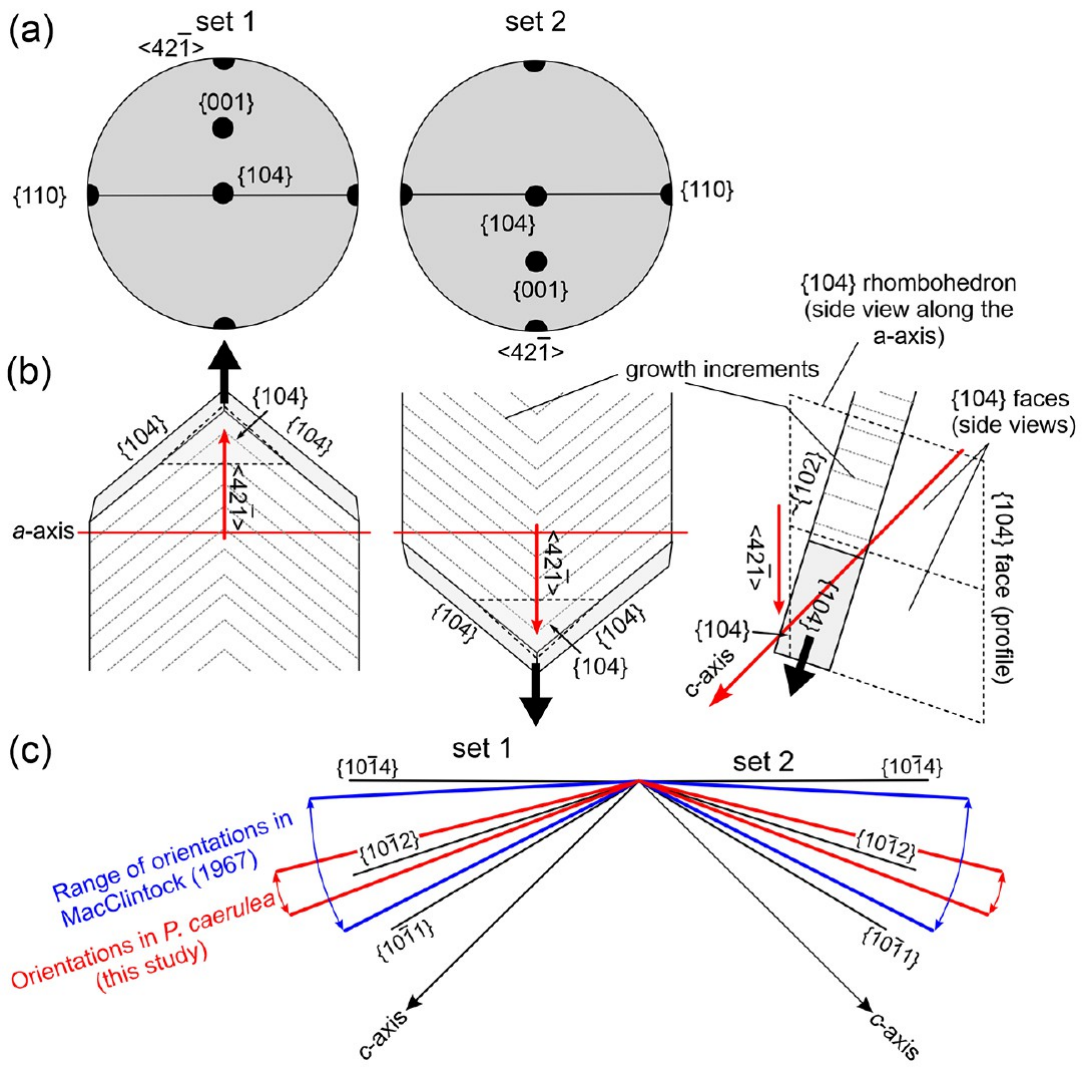
Crystallography of the CCF microstructure of Patellidae. (a) Correspondence between the distribution of maxima in idealized pole figures obtained on the growth surface (see Figure 4). (b) Distribution of crystallographic axes and faces of third OLe of alternating first OLe deduced from the idealized pole figures in (a). The lower right sketch is a side view of the lath of set 2. The {104} face looking toward the shell growth surface (in broken line) is not usually expressed. (c) Profiles of third OLe from different sources (color lines) and crystallographic faces, viewed along the *a*_2_-axis.

The pole figures obtained on the growth surfaces and in sections of the CCF layer provide congruent results, indicating that the two sets of first OLe have the same internal crystallography, although there is a change in the inclination of the *c*-axes in alternating first OLe. Figure 7a is a synthetic diagram of the distribution of maxima of the pole figures in the plane of the growth surface (Figure 4). The central 104 maximum indicates that the laths grow with one {104} plane parallel to the growth surface. This coincides with the observation of wide, smooth surfaces of the {104} type present both in the marginal thick laths observed with SEM (Figure 2d), and in those of the CCF layer under AFM (Figure S1). The approximate constancy in the position of the 104 pole figure maximum from the ICF layer to the CCF layer (Figures 3 and S2), indicates that the lamellae of the former also orient a rhombohedral {104} face parallel to the growth surface. Nevertheless, the ringlike distribution of the *a*-axis (110 pole figure) in the ICF layer is consistent with the observed disorientation of lamellae (Figure 2a–c). The placement of the common 110 maximum (Figure 7a) implies that there is an *a*-axis approximately perpendicular to the elongation of the first OL, almost coincident across the contiguous first OLe (Figure 7b).

In the CCF layer, the c-axes are contained within the planes of the first OLe and are inclined in the same direction as the second and third OLe, although at a higher angle (∼45° to the growth surface), since, according to our data, second OLe are inclined by only 14–21° with respect to the growth surface in both directions (see above and Figure 3b, bottom 001 pole figure). The angle between the growth surface (assumed to be {104} according to our crystallographic data) and the *c*-axis of calcite is ∼45° (theoretically 44.64°). Accordingly, the angle between the lath surfaces and the *c*-axis is around 24°–31° (Figure 7c). If we plot the profiles of the laths onto a view of the calcite lattice along the *a*_2_-axis and indicate the profiles of the different rhombohedral faces, the face closest to the observed range of lath inclinations is of the {102} type (Figure 7b, right sketch, and c).

MacClintock^21^ found an even higher range of inclinations, between 3° and 27° (average 13°) in 10 species of *Patella* (Figure 7c). Assuming a fixed orientation of {104} parallel to the growth surface, clearly the inclinations of the second and third OLe do not follow any particular crystallographic direction but are in a range of about 15° and 9° from {102} toward {104} and {101}, respectively (Figure 7c). These are surfaces characterized by a low density of Ca^2+^ and CO_3_^–^, which are most likely stabilized, although in an unspecific manner, by biomolecules.

The laths of the crossed-foliated microstructure are homeomorphic to those of the foliated microstructure of the bivalve orders Pectinida and Ostreida. On the contrary, the main lath surfaces are of the 10*l* type, with *l* having very high values (between 10 and 15 in oysters, and 8 and 25 in pectinids^36,37^) and not very low as in *Patella*. Foliated calcite is also found in inarticulate brachiopods of the order Craniida and in stenolaemate bryozoans. In both groups, the *c*-axis is approximately parallel to the growth axis of the laths; the orientations of the *a*-axes with respect to the main surfaces of laths are inconsistent in inarticulate brachiopods^38^, and are approximately parallel to the main surfaces in stenolaematans.^39^ Accordingly, the crystallography of the laths of the *Patella* crossed-foliated microstructure is unique.

### 4.3 Organization of the Crossed-Foliated Material

One of the most remarkable features of the calcitic layer of *Patella* is the progressive organization of the crossed-foliated material, from the exterior to the interior from both morphological and crystallographic viewpoints. Morphologically, the irregularly distributed lamellae of the ICF layer organize with growth into the concentrically arranged lamellae of the CCF layer. At the same time, the number of first OLe drastically diminishes with growth, which suggests some sort of competition between them. This is not surprising, as the first OLe of the ICF layer can be compared to the foliated grains found during the initial stages of formation of the foliated layers of oysters (Figure S5). During the formation of evenly oriented foliated layers, either smaller or less favorably oriented grains can be outcompeted.

Crystallographically, the outer ICF layer has a loose sheet texture with one single, though widely spread, maximum for the *c*-axis. Across the intermediate layer, the texture transforms into a strong sheet texture, with two individualized maxima for the *c*-axis, one for each set of first OLe (Figures 3 and S2). According to the picture provided by the pole figures, this process happens through the progressive migration of the crystallographic axes toward the positions they finally acquire in the fully developed CCF layer (Figures 5 and S4). An important aspect to consider is how the organization of the CCF takes place. One possibility is liquid crystallization. Almagro and co-workers^40^ explained the organization of the different order lamellae of the (aragonitic) crossed-lamellar microstructure, resembling the crossed-foliated, by means of liquid crystallization of chitin rods. According to them, each first OL organizes as a nematic liquid crystal domain characterized by organic rods oriented in a unique direction. Later, individual first OLe are arranged in a zebra stripe pattern, in a way similar to some solutions of semiflexible polymers.^41^ Despite the close inspection with SEM (Figure 2) and AFM (Figure S1), no organic scaffold could be found in the CCF microstructure. In addition, the organization of the CCF from the ICF layer occurs along a transitional layer, which implies a considerable time lapse.

**Figure 8:**
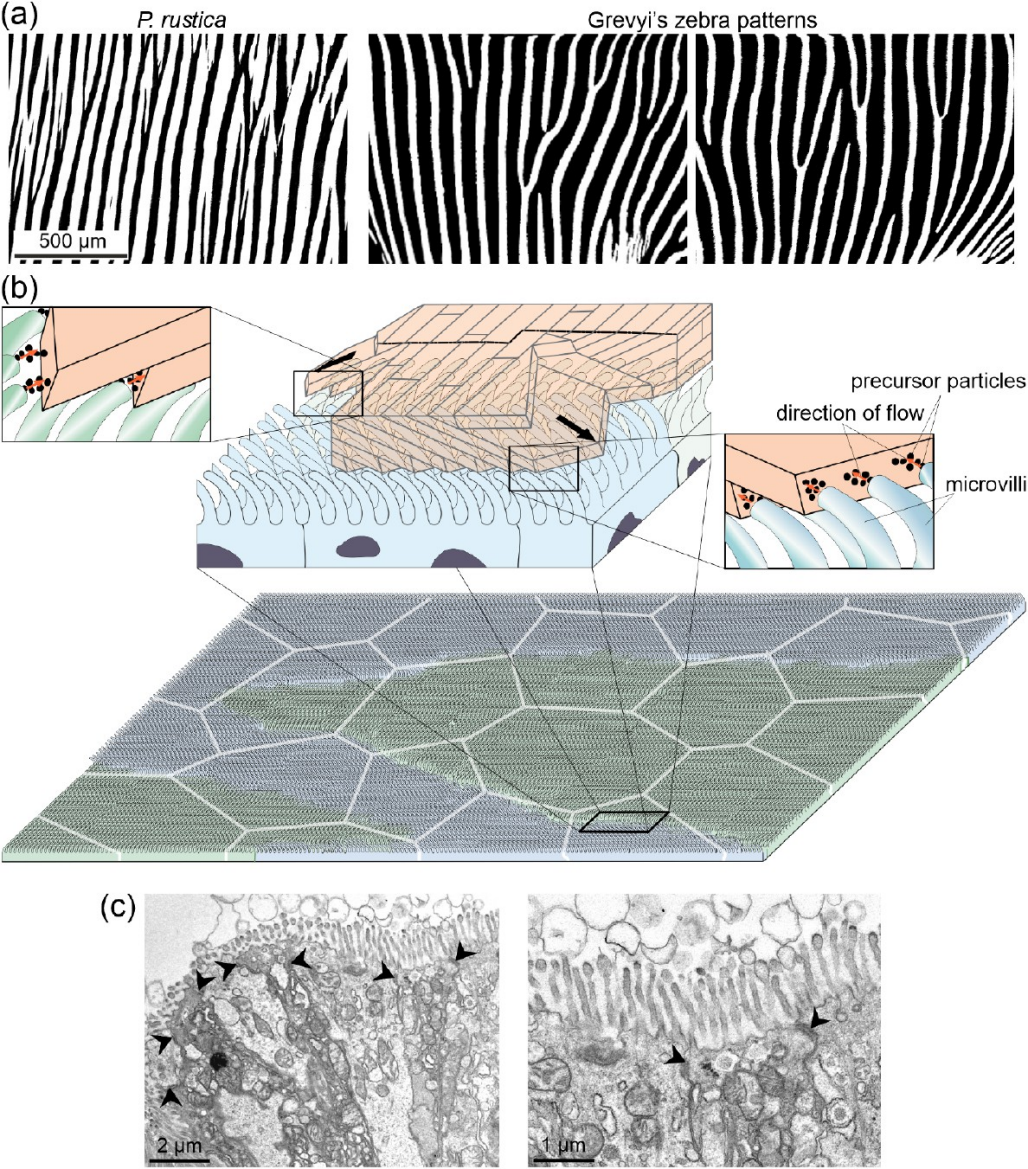
Assimilation of the first OLe of the CCF layer to a zebra pattern and hypothesis for its formation. (a) Comparison of the distribution of first OLe on the growth surface of *P. rustica* and actual zebra skin patterns. The top image is a black-and-white image of the area framed in Figure 2a. The lower images have been taken from side views of actual specimens of zebra. (b) Model for the secretion of first OLe. The zebra pattern is developed on the shell-facing mantle surface (bottom part). The cell extensions are directed sideward toward the advancing crystals, in opposite directions in alternating stripes. The growth directions of laths are guided by the opposite flows of precursor particles toward their growth surfaces. The white polygons represent the outlines of the mantle cells. (c) Two TEM images of the mantle epithelium of *P. caerulea* at different magnifications, showing the cell microvilli. They seem to be discharging vesicles to the extrapallial space. Arrows point to intercellular boundaries.

As an alternative to the liquid crystallization hypothesis, we hypothesize that the zebra patterns of the first OLe in plan view (Figures 2a and 8a) are not directly formed on the shell growth surface, but they arise first on the secretory surface of the mantle and are later somehow transferred to the shell growth surface, which it is in contact with.

Zebra patterns are relatively common in nature. While the better-known variety is that of zebra skin pigmentation, they are also found in plant leaf colors, palatal ridges in mice, the surface microplicae of skin cells from zebrafish, and others. Zebra patterns are just one variety of the many patterns derived from the reaction-diffusion kinetics developed by Turing.^42^ These patterns are produced due to the action of two morphogens (proteins or enzymes), an activator, and an inhibitor. The activator promotes the production of both morphogens, while the inhibitor acts against the production of the activator.^43^ By changing the parameters of the model (concentrations, diffusion, production, and consumption rates of the activator and the inhibitor), these two components would spontaneously self-organize into spots, rings, swirls, stripes (including the zebra type), and others.^44^ Presently, the molecular basis of many such natural patterns has been deciphered.^45^ Thus, it is conceivable that such a pattern could develop on the surface of the mantle (in fact, a “skin” made of a monocellular layer).

The next question is how the diffusion-reaction zebra pattern formed on the shell-facing mantle side translates into the zebra-like distribution of the first OLe. Here, we tentatively propose that the distribution of morphogens is expressed in different behavior of the cells belonging to alternating stripes: cells in adjacent mantle stripes curve their secretory extensions (microvilli) sideward, parallel to the elongation of the first OLe, but in opposite directions in alternating lamellae (Figure 8b). Views of the mantle cells of *P. caerulea* and their microvilli can be observed in Figure 8c. Then, microvilli extrude their precursor particles (amorphous calcium carbonate + organics) toward the advancing laths, thus, producing a flow across the thin extrapallial space, with the consequence that crystals would tend to orient in the directions of flow. It is known that, under hydrodynamic conditions, crystals are able to elongate in the direction of the ion flow due to differences in the growth rates of their faces.^46−48^ The ability to change the growth direction at constant lattice orientation has been reported in the laths of the foliated calcite of bivalves^37^ and stenolaemate bryozoans.^39^ In the CCF material, we are dealing with a polycrystalline material such that either the individual crystals can change their crystalline orientations or the more conveniently oriented laths (in parallel to the elongation of the zebra stripes) outcompete others. All things considered, we suggest that, given enough time, the components of the crossed-foliated microstructure can change their crystallographic directions progressively toward the flow of the precursor particles (Figure 8b) induced by the mantle cells. In summary, the opposite flows produced by the mantle cells would give rise to the opposite growth directions observed in the crossed-foliated material. In this way, the pattern developed on the mantle surface is transmitted to the shell growth surface. Note that in our model, the boundaries between the zebra stripes go across the individual mantle cells (usually between 5 and 10 μm in size), so that single cells may be secreting laths belonging to the alternating first OLe (Figure 8b).

## 5 Conclusions

The outer calcitic layer of the limpets of the family Patellidae is formed by crossed-foliated material. This material is hierarchically organized into first, second, and third OLe, with the latter being the basic units, consisting of individual calcitic laths with arrow point endings. They form planar arrangements (second OLe). The first OLe contain myriads of second and third OLe with similar orientations and which dip toward the shell interior.

This material organizes with growth, such that it consists initially of irregularly oriented and sized first OLe (the so-called ICF, material). With time, the number of first OLe diminishes drastically, and the first OLe acquire a commarginal orientation and similar sizes. Their arrangement on the internal shell surface can be qualified as zebra-like. This is termed as CCF material. Second and third OLe in alternating first OLe dip in opposite directions. In this way, the CCF microstructure is a plywood material.

While the ICF layer has a poor fiber texture, the CCF layer has a relatively strong sheet texture. Each set of alternating first OLe has its *c*-axis inclined in the direction of the laths of each set of first OLe, although to a higher degree. Both sets have a common {104} rhombohedral surface that is aligned with the shell growth surface, and a common *a*-axis approximately perpendicular to the elongation of the first OLe. The surfaces of laths are close in orientation to {102} calcite surfaces but do not represent actual crystallographic planes.

With the change in the arrangement of the first OLe from the ICF layer to the CCF layer, those first OLe that reach the CCF layer progressively change the orientations of their crystallographic axes to accommodate to the crystallography of the mature CCF layer.

We hypothesize that the zebra-like pattern of the first OLe of the CCF layer was developed initially in the mantle and was later transferred to the shell. How the mantle cells drive the growth of the laths (the third OLe) is unknown. We speculate that the cell microvilli produce a flow of precursor particles, which might guide the orientation of the laths.

## Supporting Information Available

AFM views of the calcite laths of the CCF layer; additional IPF maps and pole figures of the crossed-foliated layers distinguished within the shells of Patellidae; growth trajectories of selected first OLe on IPF maps; misorientation profiles across selected first OLe; foliated grains observed in the oyster *Ostrea edulis* (PDF)
