# Supplementary material for: Organization and Formation of the Crossed-Foliated Biomineral Microstructure of Limpet Shells
Source: ACS Biomater Sci Eng. 2023 Nov 22;9(12):6658–69. doi: 10.1021/acsbiomaterials.3c00928 (PMC10716850; doi:10.1021/acsbiomaterials.3c00928)
Supplement: Supplementary file 1 — ab3c00928_si_001.pdf [file ab3c00928_si_001.pdf]

Organization and Formation of the Crossed-Foliated Biomineral Microstructure of Limpet Shells

Katarzyna Berent[1], *Marta Gajewska*[1], *Antonio G. Checa*[2]

[1]Academic Centre for Materials and Nanotechnology, AGH University of Krakow, 30-059 Krakow, Poland

[2]Departamento de Estratigrafía y Paleontología, Universidad de Granada, 18071 Granada, Spain and Instituto Andaluz de Ciencias de la Tierra, CSIC–Universidad de Granada, 18100 Armilla, Granada, Spain

Supplementary Figures S1 to S5

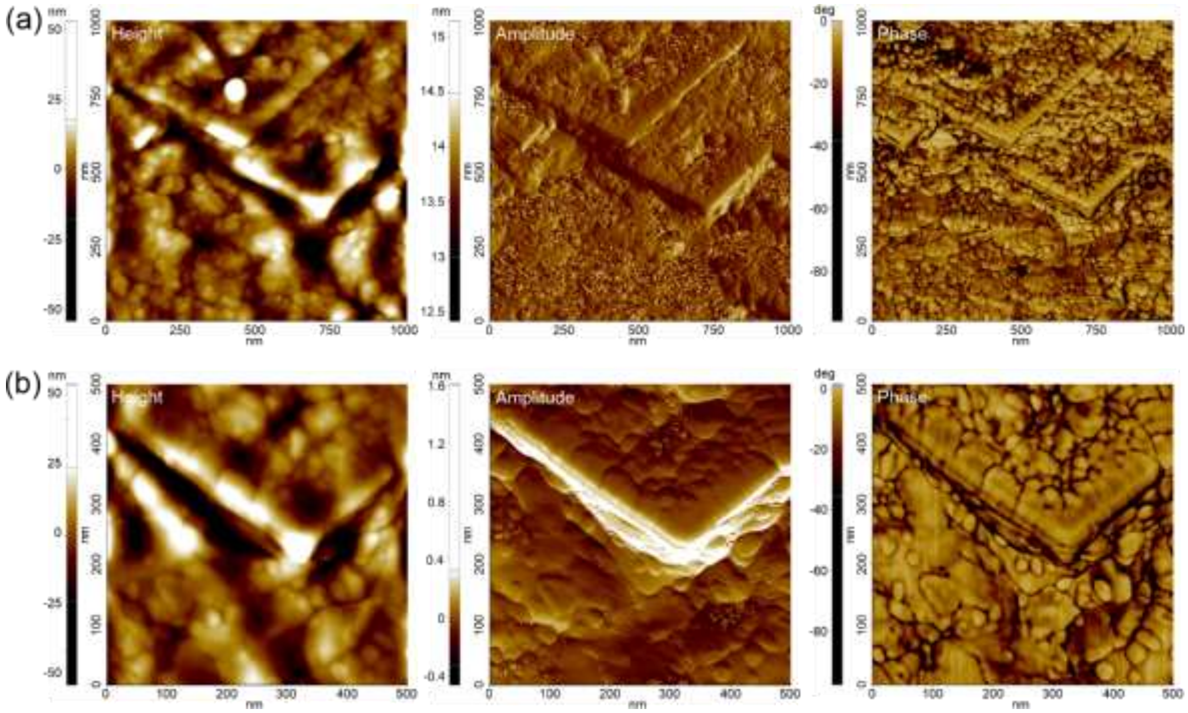

Figure S1. AFM views of the CCF layer of *P. caerulea* at two different magnifications (a, b), showing the surface nanoroughness of the laths, except for the smooth arrowhead endings. The phase images reveal the existence of a minority dark phase around the nanolumps (particularly evident in the phase image of b).

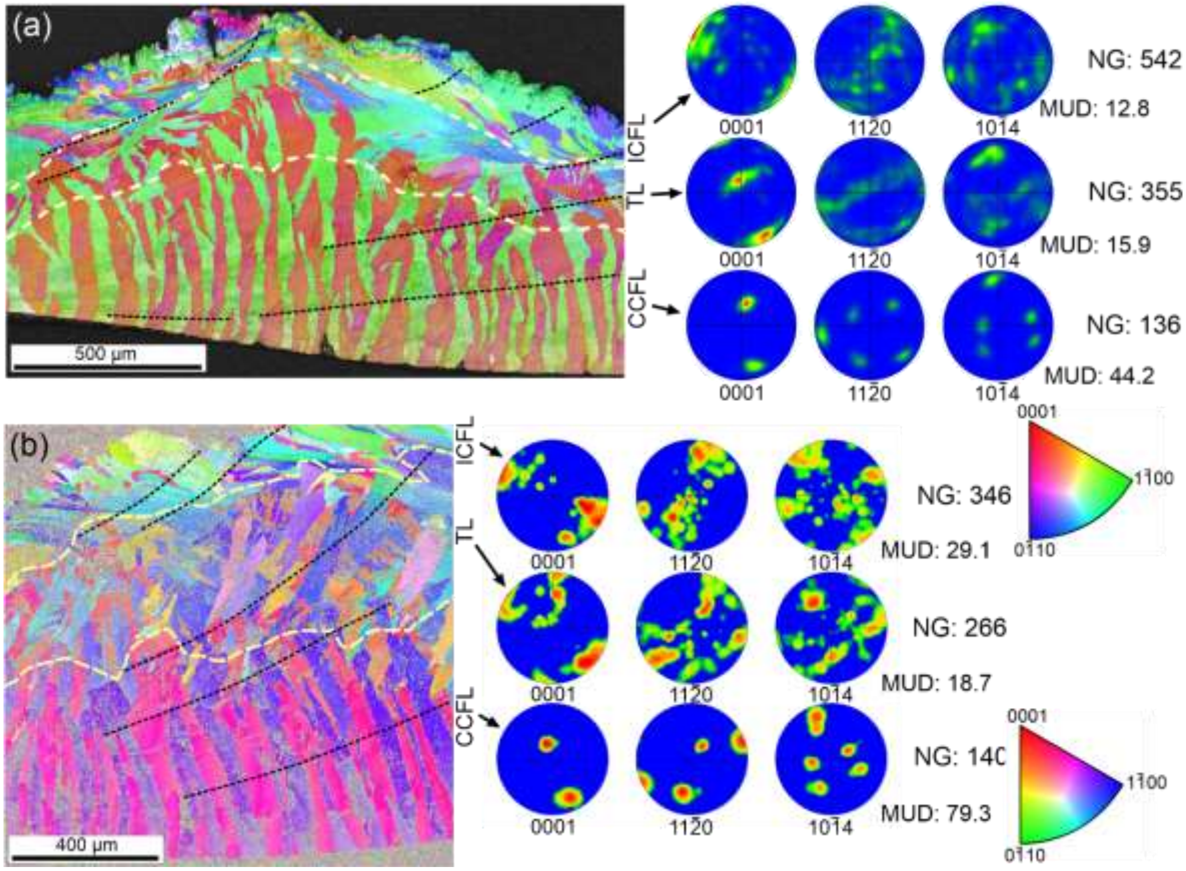

Figure S2. IPF maps and pole figures of the layers distinguished within the shells of Patellidae. (a) Radial section of P. *depressa*; growth lines are indicated with dotted lines. (b) Radial section of *P. rustica*. CCFL, ICFL, TL: concentric cross-foliated, irregular cross-foliated, transitional layers. NG is the number of grains for each layer. Color triangles are the color keys for each map.

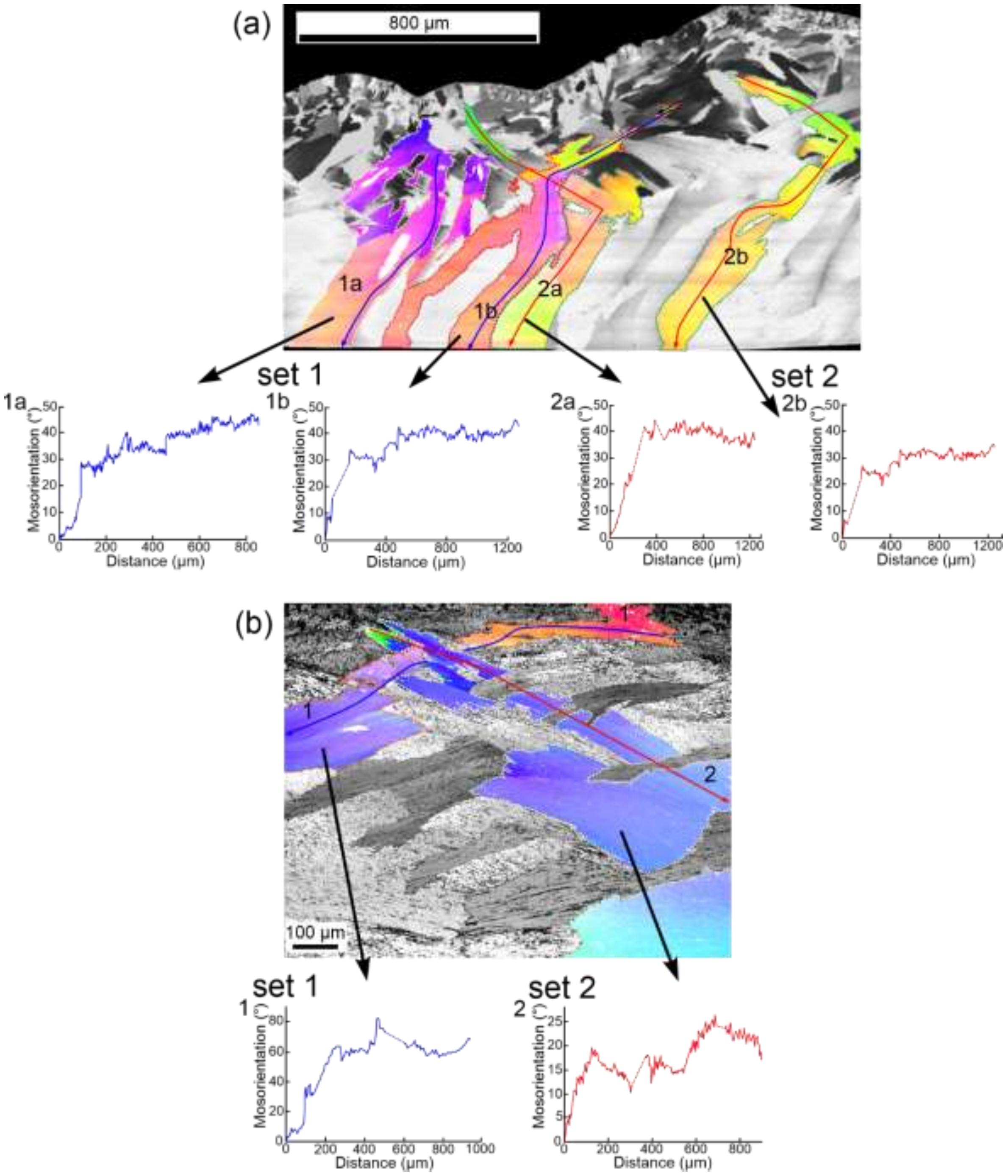

Figure S3. Misorientation profiles across the 1st OLe delineated in Figure 5. (a) *P. depressa*. (b) *P. caerulea*. As a general rule, there is a continuous, although sometimes oscillating trend. The red arrows on the lamellae are the transects along which the misorientation profiles have been plotted. Broken lines in the misorientation profiles correspond to interruptions of the transects. The color key for orientations is provided in Figure 3.

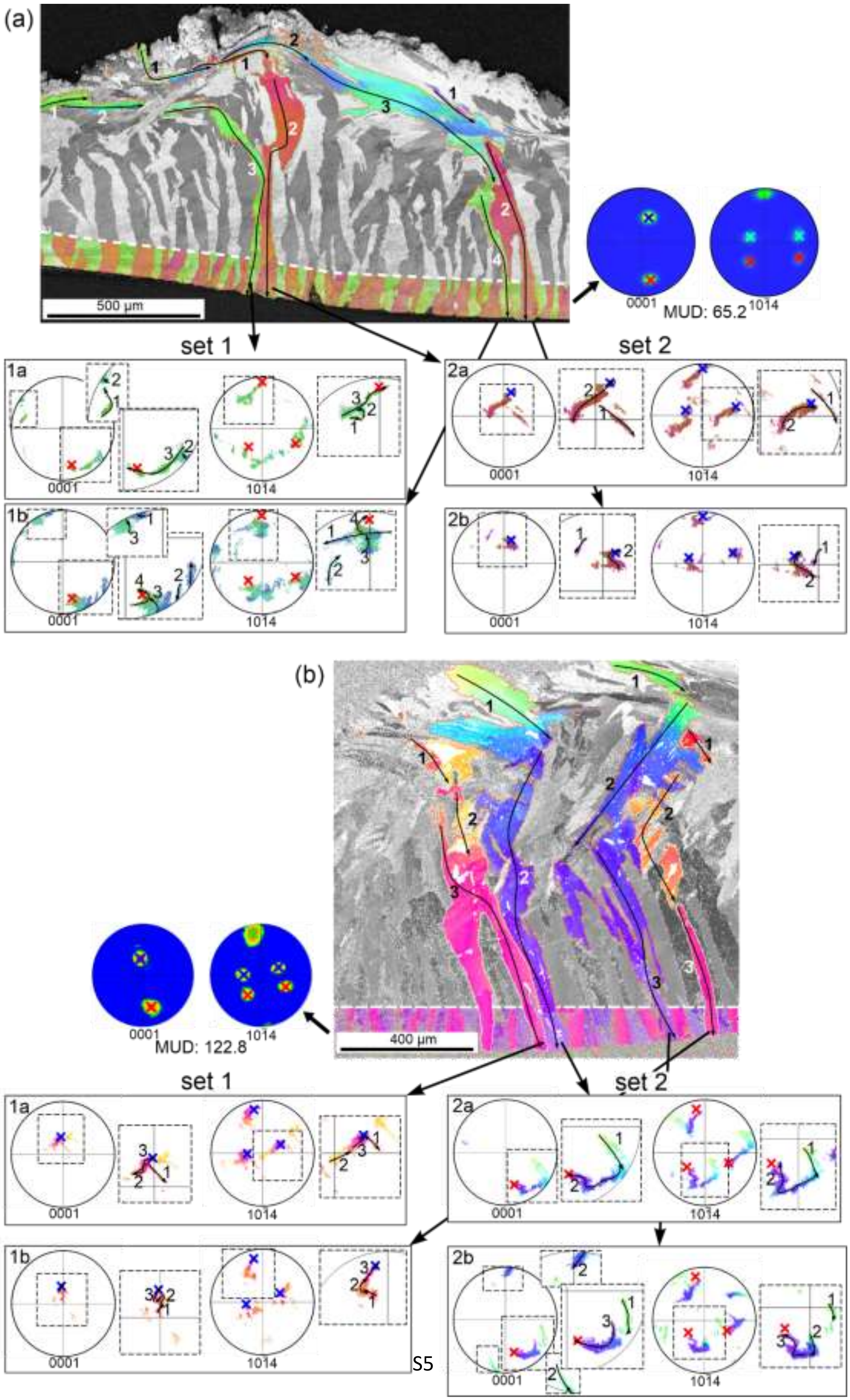
(a)
500 µm
0001
MUD: 65.2
1014
set 1
set 2
1a
1b
2a
2b
(b)
400 µm
MUD: 122.8

Figure S4. Growth trajectories of 1st OLe selected on the orientations maps of Figure S2. (a) *P. depressa*. (b) *P. rustica*. Two 1st OLe per set have been selected in both cases. Pole figures besides the orientation maps correspond to the rectangular areas close to the growth surface. Positions of maxima for the two sets of 1st OLe are indicated with red, blue, and green crosses. Individual raw pole figures for the selected lamellae are provided (color crosses indicate the positions of the maxima close to the growth surface for the corresponding set of 1st OLe). The trajectories (black arrows) are indicated both on the 1st OLe and on the magnified areas of the pole figures. These consist of 3 or 4 segments. The orientation color key for each map is provided in Figure S2.

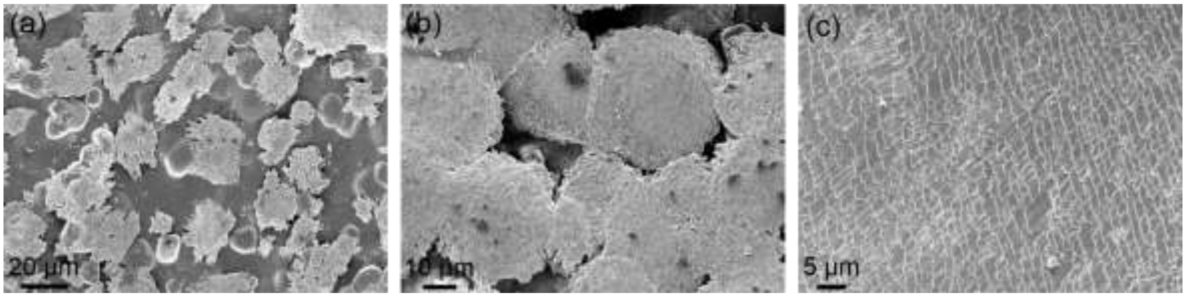

Figure S5. Foliated grains observed during the reinitiation of foliated layers in the oyster *Ostrea edulis*. (a) Grains growing onto an organic membrane, which interrupted the growth of the foliated layer. (b) Slightly more advanced stage, with grains occupying most of the organic surface. (c) Detail of the surface of one grain showing its foliated nature.
